# Supplementary material for: Comparative multiomics analysis of cell physiological state after culture in a basket bioreactor
Source: Sci Rep. 2022 Nov 23;12:20161. doi: 10.1038/s41598-022-24687-4 (PMC9686226; doi:10.1038/s41598-022-24687-4)
Supplement: Supplementary file 1 — Supplementary Information 1. [file 41598_2022_24687_MOESM1_ESM.zip › raw data/Proteomic raw data/6.KEGGenrichment/EG.vs.CG/ALL/pathway/src/00920.html]

map00920


- K17725, 1.13.11.18, R00781"

- Down regulated proteins

A0A0D9S1A5(Description:Lactamase\_B domain-containing protein;Gene:ETHE1;log2FC:-1.2277)

Close
